# Effects of a Tai Chi rehabilitation program implemented using a hybrid online and offline mode on oxidative stress and inflammatory responses in patients with coronary heart disease: a randomized controlled trial

**DOI:** 10.3389/fpubh.2024.1369675

**Published:** 2024-05-17

**Authors:** Meize Cui, Qiuyang Wei, Yameng Li, Mingyu Liu, Ligang Sun, Xinyi Liu, Zaihao Chen, Hui Fang, Linli Liu, Jiahao Fu, Cuihan Li, Haojie Li, Yuxin Ma, Xing Zhang, Yuerong Huang, Lujia Li, Shaojun Lyu, Jianwei Zhang

**Affiliations:** ^1^College of Physical Education and Sports, Beijing Normal University, Beijing, China; ^2^Sports Department, Jiangsu University, Zhenjiang, Jiangsu, China; ^3^Department of Physical Education, Northwestern Polytechnical University, Xi’an, China; ^4^University Hospital, Beijing Normal University, Beijing, China; ^5^China University of Labor Relations, Beijing, China; ^6^Zhejiang Guangsha Vocational and Technical University of Construction, Zhejiang, China

**Keywords:** Tai Chi, coronary heart disease, oxidative stress, inflammatory response, randomized controlled trial

## Abstract

**Background:**

Coronary heart disease (CHD) is the leading cause of death in both developed and many developing countries. Exercise training is a fundamental component of cardiac rehabilitation programs for patients with CHD. This study aims to investigate the effects of a Tai Chi rehabilitation program, which is provided through a hybrid online and offline mode, on oxidative stress and inflammatory responses in patients with CHD.

**Methods:**

A total of 34 patients with coronary heart disease were randomly assigned to two groups: an experiment group (*n* = 14, age 62.07 ± 9.076 years) and a control group (*n* = 20, age 61.90 ± 9.700 years). The experiment group underwent a 12-week Tai Chi cardiac rehabilitation program (TCCRP), while the control group followed a conventional exercise rehabilitation program (CERP) consisting of 1-h sessions, 3 times per week, for a total of 36 sessions. Participants were studied at baseline and post-intervention. The main assessments include the levels of Malondialdehyde (MDA), Superoxide dismutase (SOD), Tumor necrosis factor (TNF-α) and Interleukin-10 (IL − 10) in blood samples. Pearson correlation analysis was used, and the differences between the two groups were subsequently tested using two-way repeated ANOVA. Statistical significance was defined as a two-sided *p*-value of <0.05.

**Results:**

The key finding of the study reveals that MDA was significantly reduced by 1.027 nmoL/mL. Additionally, the TCCRP showed significant improvements in SOD and IL-10, with values of 10.110 U/mL and 2.441 pg./mL, respectively. Notably, a significant positive correlation was found between SOD and IL-10 (*r* = 0.689, *p* = 0.006), while MDA showed a significant positive correlation with TNF-a (*r* = 0.542, *p* = 0.045). In contrast, the ECRP group only showed a significant improvement in SOD.

**Conclusion:**

The study conducted a 12-week program on TCCRP, which utilized a hybrid online and offline model for individuals with coronary heart disease. The program showed promising results in alleviating oxidative stress and inflammation, possibly by regulating the balance between oxidative and antioxidative factors, as well as pro-inflammatory and anti-inflammatory factors.

## 1 Introduction

Coronary heart disease (CHD), also called coronary artery disease (CAD) (1), is the shortened name of coronary atherosclerotic heart disease. CHD is a common chronic disease, which is mainly due to the gradual constriction of the blood vessels that supply oxygenated blood to the myocardium (2). In recent years, studies have shown that the pathological process of coronary heart disease is closely related to oxidative stress and the inflammatory response and manifests as impaired coronary endothelial function and decreased anti-inflammatory and antioxidant functions of the body, ultimately leading to angina pectoris, myocardial infarction and other adverse events (3–6). Cardiac rehabilitation is one of the most effective strategies to mitigate the cardiovascular disease process, including lifestyle, physical activity, risk factor control, and medication guidance (7). Given that patients are easily discouraged and experience heavier burdens due treatment expenses, pressure from work or family, and the need to commute to the hospital, home cardiac rehabilitation programs based on remote online guidance have been regarded as alternatives (8, 9). Home-based cardiac rehabilitation can be performed by medical workers with remote guidance, exercise monitoring, and regular individualized feedback, but poor safety has been reported (patients may experience cardiovascular adverse events during exercise and require immediate treatment) (10). To solve the problems and limitations of hospital rehabilitation and home-based rehabilitation, hybrid online and offline rehabilitation programs have emerged based on the combination of in-hospital rehabilitation guidance and home remote online rehabilitation guidance (11). The intervention provided in this study implements a hybrid model of online and offline guidance. This model can effectively reduce the economic burden of patients, save many medical resources, provide increased flexibility, fully meet the needs of patients, and effectively improve patients’ compliance, safety, and participation.

Exercise rehabilitation is the core content of cardiac rehabilitation. Numerous studies have shown that long-term adherence to exercise rehabilitation can improve vascular endothelial function and cardiopulmonary function and reduce the recurrence rate and mortality of cardiovascular disease (12, 13). Tai Chi is a heart rehabilitation exercise program with Chinese characteristics. Studies have shown that Tai Chi can improve SOD and GSH-Px activity, reduce MDA content and improve the antioxidant capacity of patients with T2DM (14). Some studies have found that long-term and regular Tai Chi exercise can reduce the levels of proinflammatory factors, such as TNF-α and CRP, in patients with CHD and improve the endothelial microvascular function of patients (15). However, it is not clear whether Tai Chi can exert a combined antioxidant and anti-inflammatory effect and reduce oxidative-inflammatory damage to the body.

This study uses the TCCRP (16) as an intervention method and takes the typical pathological characteristics of coronary heart disease patients—oxidative stress and inflammatory response—as the breakthrough point to explore whether a Tai Chi rehabilitation program provided with mixed online and offline modes can improve the antioxidant and anti-inflammatory capacity of heart disease patients compared to conventional exercise rehabilitation regimens. The authors hope to provide a more convincing scientific basis for the clinical benefits of the TCCRP and build a individualized, safe, effective, and suitable cardiac exercise rehabilitation program for coronary heart disease.

## 2 Methods

### 2.1 Study design

This study is a randomized controlled clinical trial. The participants were randomly assigned into two groups (1:1 ratio): an experimental group (Tai Chi Cardiac Rehabilitation Program, TCCRP) and a control group (conventional exercise rehabilitation Program, CERP). The research lasted for a total of 12 weeks. The process of the study involved recruiting participants, screening them, randomly assigning them to groups, implementing the intervention, and conducting follow-up assessments. Following the ethical guidelines outlined in the Declaration of Helsinki, all subjects were provided with comprehensive information regarding the exercise program before providing their signature on the informed consent form.

This study was approved by the Ethics Committee of the Chinese People’s Liberation Army General Hospital and registered with the International Clinical Trials Registry (Clinical Trials.gov PRS) (Approval number: S2019-060-02) (Registration number: NCT03936504).

### 2.2 Participants

**Figure 1:**
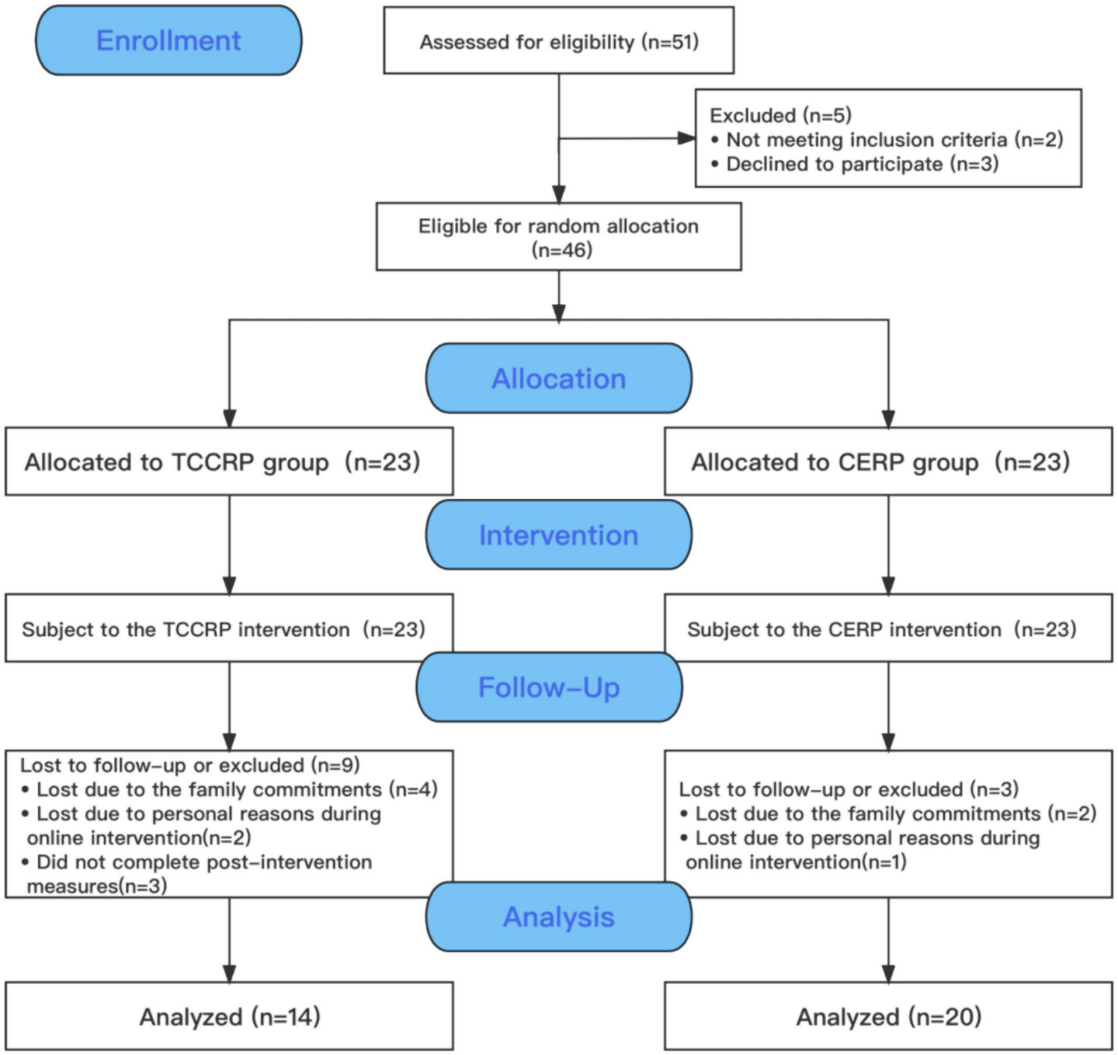
Flow of participants through the study. TCCRP, Tai Chi cardiac rehabilitation program; CERP, Conventional exercise rehabilitation program. The reasons of did not complete post-intervention measures: due to the travel cannot reach the test point on time (*n* = 2); did not provide the reason (*n* = 1).

From October 2020 to November 2021, a total of 46 patients with CHD were eligible to participate in the study at Anzhen Community Health Service Center, Chaoyang District, Beijing, and Wanjie Rehabilitation Hospital, Zibo, Shandong. To randomly allocate the participants into the Tai Chi Cardiac Rehabilitation Program (TCCRP group) or conventional exercise rehabilitation program (CERP group). The flow of the participants through the study is presented in Figure 1. Twenty did not complete the 12-week interventions with pre-and post-intervention assessments. Following assessment based on the criteria for inclusion and exclusion, only 34 CHD patients were retained in the sample. Including 14 in the TCCRP group with an average age of 62.07 ± 9.076 years. There were 20 cases in the CERP group with an average age of 61.90 ± 9.700 years. Prior to each exercise intervention, therapists conducted a daily health questioning and assessment of the patients, which included smoking, alcohol consumption, sleep, diet, and emotional well-being.

All patients who met all inclusion and exclusion criteria and were granted written informed consent to participate were entered into the study. The inclusion criteria: ① Age and sex: male or nonpregnant female aged between 30 and 80 years; ② Cardiac function classification: NYHA class I and II; ③ Diagnostic criteria: met the diagnostic criteria for chronic coronary syndrome based on the “2019 ESC Guidelines for the Diagnosis and Management of Chronic Coronary Syndrome (CCS)” issued by the European Society of Cardiology (ESC) in 2019; ④ subjects need to understand the purpose of the clinical trial, participate voluntarily and sign the informed consent form.

The exclusion criteria were as follows: ①acute myocardial infarction within 2 weeks; ②severe aortic stenosis; ③hypertrophic cardiomyopathy; ④severe valvular heart disease; ⑤malignant tachyarrhythmia; and ⑥patients with abnormal motor function caused by neurological deterioration, motor system disease or rheumatism.

### 2.3 Sample size calculation

We performed an a prior power analysis based on previous research (17) using G*Power V.3.1 (18) for the MDA levels between comparison groups with a 5% significance level, employing a two-tailed critical region to identify an effect size of Cohen’s *d* = 0.50 with 80% power. According, it needs at least 32 participants. Lastly, we included about 46 older person with CHD (the study was conducted during the midst of a Covid-19 pandemic, so we set a 30% dropout) from community hospitals.

### 2.4 Intervention modes and intervention programs

#### 2.4.1 Intervention modes

This study adopts a hybrid online and offline model combining hospital rehabilitation guidance and home-based remote online rehabilitation guidance. First, the patients with coronary heart disease received 4 weeks of hospital rehabilitation guidance. Then, patients were guided and supervised for 8 weeks of home-based rehabilitation via a Tencent online conference. All subjects have a sitter or a care-giver at home to monitor them at home. The use of face-to-face rehabilitation guidance in the hospital and home-based remote online real-time rehabilitation guidance can effectively reduce the economic burden on patients and save many medical resources, providing more flexibility to fully meet the needs of CHD patients during the COVID-19 pneumonia epidemic and effectively improve patient compliance, safety, and engagement.

Before the intervention, a unified epidemiological questionnaire was used to inquire about and record the general conditions of the patients, including smoking and drinking history, previous medical history, family medical history, and medication history. In addition, the body composition analyzer Inbody 720 (Biospace) was used to measure demographic parameters, such as height, weight, BMI, and body fat percentage. Biochemical indicators of oxidative stress and the inflammatory response were measured before and after the experiment.

#### 2.4.2 Intervention programs

The intervention lasted 12 weeks (4 weeks of hospital rehabilitation guidance and 8 weeks of home-based remote online real-time rehabilitation guidance). A cardiopulmonary exercise testing (CPET) was performed by patients to obtain information on their exercise tolerance before intervention. According to the exercise testing guidelines recommended by the American College of Cardiology (ACC) and the American College of Sports Medicine (ACSM), the cardiopulmonary exercise test in this study utilized a graded ascending exercise protocol (Ramp protocol), with a total exercise test time expected to fall within 8–12 min (19, 20). The termination criteria included reaching the target heart rate, as well as monitoring for various cardiac abnormalities such as frequent ventricular premature contractions, polygenic ventricular premature contractions, ventricular tachycardia, paroxysmal supraventricular tachycardia, ventricular fibrillation, slow heart rate, angina, decreased blood pressure, dyspnea, shortness of breath, fatigue, inability to persist, and ischemic ST segment depression (21).

Then, professional physicians formulated exercise prescriptions for the two groups of patients based on the results of cardiopulmonary exercise tests, echocardiograms, past medical history, and medication history (22, 23). The specific exercise intervention plan is shown in Table 1.

**Table 1:** Intervention plan.

Content	Conventional exercise rehabilitation program group	Tai Chi cardiac rehabilitation program group
Program	Conventional exercise rehabilitation program	Tai Chi cardiac rehabilitation program
Stage	4 weeks (in-hospital rehabilitation) + 8 weeks (home online rehabilitation)	4 weeks (in-hospital rehabilitation) + 8 weeks (home online rehabilitation)
Frequency	Three times per week for 12 weeks	Three times per week for 12 weeks
Time	Warm-up exercises (10 min) + aerobic activity (30 min) + elastic belt resistance exercise (10 min) + relaxed stretching (10 min)	Tai Chi warm-up exercises (10 min) + Bafa Wubu of Tai Chi (30 min) + Tai Chi elastic belt exercise (10 min) + Tai Chi cool-down exercises (10 min)
Intensity	40–60% heart rate reserve combined with an anaerobic threshold method; rating of perceived exertion (11–13)	40–60% heart rate reserve combined with an anaerobic threshold method; rating of perceived exertion (11–13)
Notes	Prior preparation of water and energy supplies required for exercise. Monitoring the subject’s heart rate, blood pressure, and rating of perceived exertion before, during, and after exercise. Prohibition of breath-holding during exercise. Exercise performed in a gradual manner and with their own safety in mind.	

The exercise intensity corresponds to 40–60% heart rate reserve combined with an anaerobic threshold method. Age-predicted peak exercise heart rate was calculated using the formula: 208–(0.7age).

#### 2.4.3 Safety supervision and precautions

① During the exercise intervention, patients wore an MIO heart rate monitoring smart bracelet (MIO FUSE Heart Rate Training), which is a real-time heart rate monitor. Changes in blood pressure, blood oxygen, and heart rate were monitored by accompanying staff and medical staff before, during, and after each exercise. In addition, the rated perceived exertion (RPE) was used to evaluate the subjective feelings of patients during and after exercise.

② Patients with unstable conditions must be accompanied and monitored by their family members during the recovery process at home.

③ During the entire experimental period, the patients’ physical activity level and adverse events during the period were monitored according to the rehabilitation record sheet. The physical activity level of patients was classified as less than 3 times per week, greater than or equal to 3 times per week but less than or equal to 5 times per week, and greater than 5 times per week.

### 2.5 Blood sample collection and evaluation

Blood collection: briefly, 5 mL of peripheral blood (including an anticoagulant tube with separating gel) was obtained from patients on an empty stomach in the morning 1 week before and 1 week after exercise rehabilitation. Samples are allowed stand for 1–2 h for coagulation and stratification followed by centrifugation at 3000 rpm for 10 min at low temperature. After separation, serum was collected, and the serum was divided into EP tubes for testing.

Required instruments: visible spectrophotometer, DR-200BS enzyme label analyzer, A6 semiautomatic biochemical analyzer, centrifuge, adjustable pipette, EP tube, constant temperature water bath box, and distilled water.

Evaluation of the oxidative stress index: Oxidant: malondialdehyde is the product of lipid oxidation (LPO) damage, and the concentration of MDA reflects the degree of lipid peroxidation and the level of oxygen free radicals in the body. Antioxidant: SOD belongs to the class of antioxidant enzymes, and its activity reflects the body’s ability to scavenge oxygen-free radicals. Assay methods: Blood collection, anticoagulation, and centrifugation were performed according to the kit instructions. The concentrations of malondialdehyde (MDA) and superoxide dismutase (SOD) were determined by spectrophotometry. The item numbers are HY-M003 and HY-M0001.

Evaluation of inflammatory response index: Proinflammatory factors: tumor necrosis factor (TNF-a) is used as an indicator to reflect the proinflammatory response of the patient’s body. Anti-inflammatory factor: interleukin 10 (IL-10) is used as an indicator to reflect the anti-inflammatory effect of the patient’s body. Assay method: blood collection, anticoagulation, and centrifugation were performed according to the kit instructions. Tumor necrosis factor (TNF-a) and interleukin 10 (IL-10) were detected using an enzyme-linked immunosorbent assay (ELISA), and the kits were provided by a biotechnology research.

### 2.6 Randomization and blinding

This study randomized the participants into two groups using the simple randomization method. The random allocation sequence will be produced by an independent statistician via the PLAN sentences of the statistical software SAS V.9.2 in a 1:1 ratio.

Next, these assignments will be sent to a study staff member, exclusive to the study coordinator or principal investigator, who will store them into sealed, opaque envelopes with date and signature labels placed over the seals of the envelopes. The randomization envelopes will not be opened unless a participant meets eligibility criteria, completes the informed consent and undergoes a baseline assessment.

Blindfolding the participants was difficult because of the characteristics of the intervention measures. Hence, only indicator testers and data analysts were blindfolded.

### 2.7 Data analysis

In this study, per-protocol (PP) analysis was employed. Data analysis was conducted by using SPSS 29.0 (IBM SPSS Statistics for Windows, USA). All data were checked for outlier’s presence, normal distributions (Kolmogorov–Smirnov test), and homogeneity of variance (Levene’s test). Descriptive statistics were computed for all characteristic variables at baseline. Moreover, pre-intervention differences in the variables between groups were examined to test whether any clinical or anthropometric items could be added as a covariate in the analysis of variance (ANOVA). Two-way repeated measures ANOVA was used to analyze the effects of oxidative stress and inflammatory response indexes before and after the intervention in the two groups and the interaction between them. Pearson correlation analysis was used to analyze the correlation between changes in oxidative stress and inflammatory response indexes. Data are expressed as mean ± SD unless otherwise stated, and categorical variables are described as frequency. Statistical significance was defined as a two-sided *p*-value of <0.05.

## 3 Results

### 3.1 Baseline characteristics of participants

Before the intervention, there were no significant differences (*p* > 0.05) in any of the variables were found between the two groups. Baseline values of the characteristics are reported in Table 2.

**Table 2:** Basic information on patients with CHD in the two groups.

Items	TCCRP group (*n* = 14)	CERP group (n = 20)	Statistical coefficient	*p-*value
Gender				
Male, *n* (%)	12 (85.7)	14 (70.0)	1.130^b^	0.422
Female, *n* (%)	2 (14.3)	6 (30.0)		
Age	62.07 ± 9.076	61.90 ± 9.700	0.052^a^	0.959
SBP (mmHg)	119.71 ± 16.582	120.50 ± 19.880	0.122^a^	0.830
DBP (mmHg)	75.07 ± 9.401	76.35 ± 11.918	0.335^a^	0.340
BMI (kg/m2)	26.65 ± 2.926	26.090 ± 2.524	0.596^a^	0.555
LVEF (%)	62.778 ± 4.902	61.200 ± 5.970	0.815^a^	0.948
MDA (nmol/ml)	4.929 ± 1.052	4.230 ± 0.990	1.942^a^	0.057
SOD (U/ml)	43.594 ± 9.888	49.958 ± 10.669	−1.763^a^	0.087
IL-10 (pg/ml)	13.168 ± 1.709	14.659 ± 2.852	−1.745^a^	0.091
TNF-a (pg/ml)	77.622 ± 16.259	79.996 ± 15.982	−0.432^a^	0.675
Smoking history				
Never smoke, *n* (%)	6 (42.86)	10 (50.00)	0.169^b^	0.738
Used to smoke, now quit, *n* (%)	8 (57.14)	10 (50.00)		
Currently smoking, *n* (%)	0	0		
Drinking history				
Never drink, *n* (%)	4 (28.57)	11 (55.00)	2.358^b^	0.308
Used to drink, now quit, *n* (%)	3 (21.43)	3 (15.00)		
Currently drinking, *n* (%)	7 (50.00)	6 (30.00)		
Type of revascularization				
PCI, *n* (%)	7 (50.00)	7 (25.00)	0.765^c^	0.487
CABG, *n* (%)	1 (7.14)	4 (20.00)	1.085^c^	0.379
None, *n* (%)	6 (42.86)	9 (45)	0.169^c^	0.738
Combined disease				
Hypertension, *n* (%)	10 (71.43)	14 (70.00)	0.008^c^	1.000
Diabetes, *n* (%)	3 (21.43)	6 (30.00)	0.311^c^	0.704
Hyperlipemia, *n* (%)	9 (64.29)	15 (75.00)	0.455^c^	0.704
None, *n* (%)	2 (14.29)	1 (5.00)	0.883^c^	0.555
Combination medication				
Antiplatelet drugs, *n* (%)	13 (92.86)	15 (75.00)	1.807^c^	0.364
Beta-blockers, *n* (%)	9 (64.29)	13 (65.00)	0.002^c^	1.000
Statins, *n* (%)	11 (78.57)	18 (90.00)	0.858^c^	0.627
Nitrates, *n* (%)	5 (35.71)	10 (50.00)	0.682^c^	0.495
ACEI/ARB, *n* (%)	4 (28.57)	5 (25.00)	0.054^c^	1.000

^a^*t*-value; ^b^Chi-square test; ^c^Fisher’s exact test; SBP, systolic blood pressure; DBP, diastolic blood pressure; BMI, body mass index; LVEF, left ventricular ejection fraction; MDA, malondialdehyde; SOD, superoxide dismutase; IL-10, interleukin 10; TNF-a, tumor necrosis factor; PCI, percutaneous coronary intervention; CABG, coronary artery bypass grafting; ACEI, angiotensin-converting enzyme inhibitor; ARB, angiotensin II receptor blocker.

### 3.2 Comparison of oxidative stress in the two groups

As shown in Table 3, Compared to the change in SOD, there was no interaction between group and time [*F*(1, 32) = 3.084, *p* = 0.089, *η*2 = 0.088]. The change in SOD had a main effect of time (*F* = 23.740, *p* < 0.001, *η*2 = 0.426). Group-by-time interactions were significantly different on the MDA [*F*(1, 32) = 8.258, *p* = 0.007, *η*2 = 0.205]. The simple effect test showed that the change in MDA had a main effect of time (*F* = 20.340, *p* < 0.001, *η*2 = 0.610).

**Table 3:** Two-way repeated measures ANOVA of oxidative stress and inflammation factors.

Indicators	Group			Time			Group*time		
MDA (nmol/ml)	*F*	*p*	*η*2	*F*	*p*	*η*2	*F*	*p*	*η*2
	1.507	0.241	0.104	20.340	<0.001**	0.610	8.258	0.007**	0.205
SOD (U/ml)	*F*	*p*	*η*2	*F*	*p*	*η*2	*F*	*p*	*η*2
	0.995	0.336	0.029	23.740	<0.001**	0.426	3.084	0.089	0.088
IL-10 (pg/ml)	*F*	*p*	*η*2	*F*	*p*	*η*2	*F*	*p*	*η*2
	1.778	0.192	0.053	10.069	0.003**	0.239	0.277	0.602	0.009
TNF-a (pg/ml)	*F*	*p*	*η*2	*F*	*p*	*η*2	*F*	*p*	*η*2
	0.858	0.361	0.026	3.676	0.064	0.103	0.471	0.498	0.014

*p < 0.05, **p < 0.01. F, Levene’s Test for Equality of Variances; *η*2, Partial Eta Squared. MDA, malondialdehyde; SOD, superoxide dismutase; IL-10, interleukin 10; TNF-a, tumor necrosis factor.

Compared with before intervention, MDA in the TCCRP group was significantly reduced after intervention (4.929 ± 1.052 vs. 3.901 ± 0.729, *p* < 0.01), while there was no significant difference in the CERP group (*p* > 0.05); SOD in the TCCRP group was significantly increased after intervention (43.594 ± 9.888 vs. 53.704 ± 11.577, *p* < 0.01). In addition, the CERP group reported a significantly increased after intervention (49.958 ± 10.669 vs. 54.711 ± 13.656, *p* < 0.05). The results are presented in Table 4.

**Table 4:** Comparison of the oxidative stress and inflammation factors of the two groups (M ± SD).

Indicators	TCCRP group(*n* = 14)		CERP group(*n* = 20)	
	Baseline	Week 12	Baseline	Week 12
MDA (nmol/ml)	4.929 ± 1.052	3.901 ± 0.729**	4.230 ± 0.990	3.933 ± 1.185
SOD (U/ml)	43.594 ± 9.888	53.704 ± 11.577**	49.958 ± 10.669	54.711 ± 13.656*
IL-10 (pg/ml)	13.168 ± 1.709	15.608 ± 2.970*	14.659 ± 2.852	16.256 ± 3.830
TNF-a (pg/ml)	77.622 ± 16.259	67.942 ± 25.011	79.996 ± 15.982	75.418 ± 17.424

*p < 0.05, **p < 0.01, after intervention vs. before intervention; ^#^*p* < 0.05, comparison between the 2 groups, TCCRP vs. CERP. MDA, malondialdehyde; SOD, superoxide dismutase; IL-10, interleukin 10; TNF-a, tumor necrosis factor.

### 3.3 Comparison of inflammation factors in the two groups

As shown in Table 3, compared to the change in IL-10 and TNF-a, there were no interaction between group and time[IL-10: *F*(1, 32) = 0.277, *p* = 0.602, *η*2 = 0.009; TNF-a: F(1, 32) = 0.471, *p* = 0.498, *η*2 = 0.014]. The change in IL-10 had a main effect of time(*F* = 10.069, *p* = 0.003, *η*2 = 0.239). Compared with before intervention, IL-10 in the TCCRP group was significantly increased after intervention (13.168 ± 1.709 vs. 15.608 ± 2.970, *p* < 0.01), while there was no significant difference in the CERP group (*p* > 0.05). No statistical difference was identified in TNF-a at W12 in two groups (*p* > 0.05), see Table 4.

### 3.4 Correlation analyses between oxidative stress and the inflammatory factors after TCCRP intervention

In the next step of the analyses we decided to analyze the correlation between oxidative stress and the inflammatory factors after the intervention. The correlation coefficients of MDA-SOD and IL-10-TNF-a were calculated using Pearson correlation analysis. A significant positive correlation was found between SOD and IL-10 (*r* = 0.689, *p* = 0.006), while MDA showed a significant positive correlation with TNF-a (*r* = 0.542, *p* = 0.045), see Table 5.

**Table 5:** Correlation between MDA-SOD and IL-10-TNF-a after intervention.

Indicators	MDA (nmol/ml)		SOD (U/ml)	
	*r*	*p*	*r*	*p*
IL-10 (pg/ml)	−0.458	0.100	0.689**	0.006
TNF-a (pg/ml)	0.542*	0.045	−0.492	0.074

MDA, malondialdehyde; SOD, superoxide dismutase; IL-10, interleukin 10; TNF-a, tumor necrosis factor. **p < 0.01, *p < 0.05.

## 4 Discussion

This study aimed to confirm the impact of a 12-week regular Tai Chi rehabilitation program on oxidative stress and inflammatory factors in older person patients with CHD in the community. The main findings revealed that the comprehensive exercise program conducted over 12 weeks resulted in improved levels of lipid peroxidation MDA, antioxidant enzyme SOD, and anti-inflammatory factor IL-10 in patients with coronary heart disease. However, no significant difference was observed in tumor necrosis factor levels.

Previous studies have demonstrated the beneficial effects of exercise on the prognosis of patients with CHD (24). It is widely accepted that oxidative stress and inflammation are key factors in the progression and pathogenesis of CHD (25). As the main indicators regulating the balance between oxidation and antioxidation in the body, MDA and SOD play an important role in inhibiting atherosclerosis (26). The more severe the proinflammatory and anti-inflammatory imbalance of the body in patients with coronary heart disease, the more biased the proinflammatory direction is, the less stable the atherosclerotic platelets are, and the more likely cardiovascular events will occur (27).

The results of this study showed that the TCCRP significantly increased the activity of antioxidant enzymes and reduced the level of lipid oxides in patients with coronary heart disease, exerting antioxidant effects. In addition, the CERP also had positive antioxidant effects. The TCCRP could significantly exert anti-inflammatory effects, whereas the CERP failed to exert significant anti-inflammatory effects. This finding indicates that the TCCRP is conducive to reducing oxidative stress and inflammation in the patient’s body and can significantly improve the antioxidant and anti-inflammatory capacity of the patient’s body. To investigate the correlation between oxidative stress and the inflammatory response after intervention with the TCCRP, correlation analysis was performed for each index of oxidative stress and the inflammatory response. It was observed that the antioxidant enzyme SOD was significantly correlated with the change in the anti-inflammatory factor IL-10 (Table 5). The oxidant MDA was significantly correlated with the change in the proinflammatory factor TNF-a (Table 5). This fin7ding indicates that oxidative stress and the inflammatory response in the TCCRP group interacted with each other to exert antioxidant and anti-inflammatory effects.

To delve into the reasons behind this finding, this paper starts from two assumptions. On the one hand, the antioxidant effect of the TCCRP may result from the abdominal breathing method that promotes “deep, even, thin and long” breaths. This method enhances the intensity and depth of breathing without increasing the respiratory rate, thereby improving the oxygen uptake and utilization capacity of skeletal muscle in patients with coronary heart disease and promoting the antioxidant effect of the body. It also promotes adaptive changes in the body’s antioxidant system, regulates the balance of oxidants and antioxidants, and exerts antioxidant effects.

On the other hand, the TCCRP exerts anti-inflammatory effects probably due to the balance of the sympathetic-parasympathetic system and the anti-inflammatory effects exerted by the neuroendocrine-immune pathway (HPA) (28). It has been suggested that Tai Chi can effectively inhibit the release of proinflammatory factors by inhibiting sympathetic nerves (adrenergic) and activating parasympathetic nerves (cholinergic) to achieve the balance of the sympathetic-parasympathetic system. Tai Chi may also activate the afferent branch of the vagus nerve through neuro-endocrine-immune pathways that promote endogenous glucocorticoids (GC), the end product of hypothalamic–pituitary–adrenal (HPA) pathway, to produce powerful anti-inflammatory effects (29).

**Figure 2:**
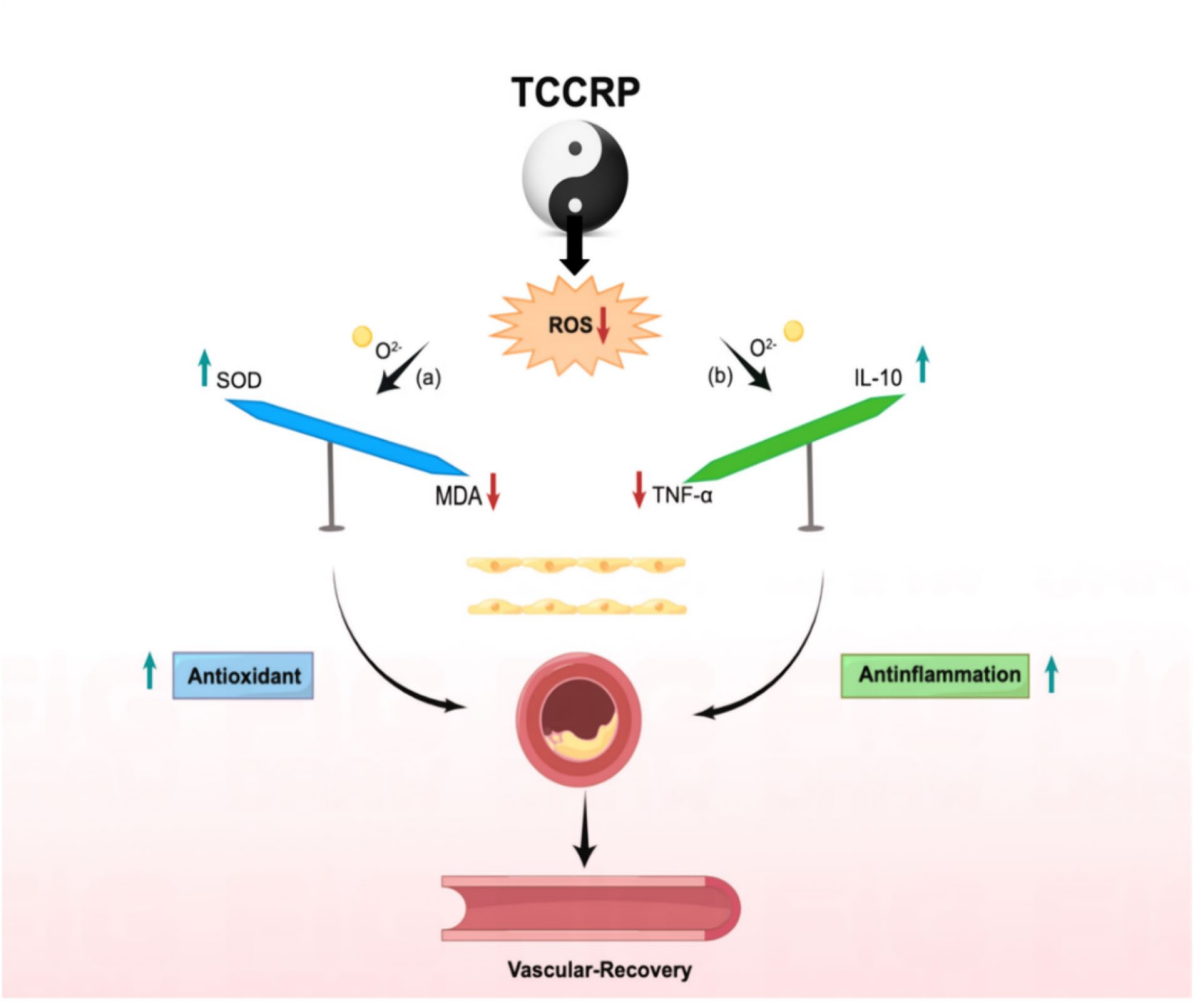
Combined antioxidant and anti-inflammatory activity of Tai Chi rehabilitation program for patients with coronary heart disease. MDA, malondialdehyde; SOD, superoxide dismutase; IL-10, interleukin 10; TNF-a, tumor necrosis factor.

The TCCRP in this study advocates the coordination of Yi, Qi, and Form during the intervention process and maintains a soft and slow breathing rhythm. Lower breathing frequency can reduce sympathetic nerve activity and enhance vagal tone (30), which is conducive to improving the balance of the sympathetic-parasympathetic system, reducing oxidative stress and inflammatory damage (31), and exerting combined antioxidant and anti-inflammatory effects. The TCCRP improves vascular homeostasis in patients with coronary heart disease through combined antioxidant and anti-inflammatory effects, see Figure 2 (By Figdraw).

This study innovatively demonstrates that Tai Chi exercise exerts a combined antioxidant and anti-inflammatory effect on patients with coronary heart disease, and the combined online and offline interventions can provide empirical support for the implementation and promotion of Tai Chi and other traditional sports and health practices in the field of cardiac rehabilitation. Our study also utilizes modern remote network and electronic information technology to promote the implementation of Tai Chi rehabilitation programs in hospitals and homes. Compared with CERP, the TCCRP is more cost-effective and convenient. Thus it is recommended to train community workers or sports instructors to implement a comprehensive Tai Chi exercise program for older person patients with coronary heart disease in the community, enriching the existing forms of cardiac exercise rehabilitation.

To better understand the influence of exercise rehabilitation on the health of patients with CHD, it is important to acknowledge the limitations of this study and take necessary steps. First, this study is exploratory, and practical problems, such as older patient age, various modes of blood vessel reconstruction, irregular medication history, and individual variability, are noted. Thus, we will expand the sample size and extend the intervention period to verify the clinical benefits of Tai Chi for patients with coronary heart disease.

## 5 Conclusion

A 12-week program on TCCRP was conducted, using a hybrid online and offline model for individuals with coronary heart disease. The program demonstrated promising results in reducing oxidative stress and inflammation, potentially through the regulation of the balance between oxidative and antioxidative factors, as well as pro-inflammatory and anti-inflammatory factors.

## Data availability statement

The datasets presented in this article are not readily available because the raw data supporting the conclusion of this article will be made available by the authors, without undue reservation. Requests to access the datasets should be directed to JZ, 13161615533@163.com.

## Ethics statement

The studies involving humans were approved by Chinese People’s Liberation Army (PLA) General Hospital. The studies were conducted in accordance with the local legislation and institutional requirements. The participants provided their written informed consent to participate in this study. Written informed consent was obtained from the individual(s) for the publication of any potentially identifiable images or data included in this article.

## Author contributions

MC and QW: Writing – original draft, Writing – review & editing. SL: Conceptualization, Methodology, Writing – review & editing. JZ: Funding acquisition, Investigation, Writing – review & editing. YL: Methodology, Project administration, Supervision, Writing – review & editing. ML: Investigation, Supervision, Writing – review & editing. LS and XL: Supervision, Resources, Writing – review & editing. ZC, HF and LiL: Investigation, Writing – review & editing. CL, HL, YM, YH and LuL: Supervision, Writing – review & editing. JF and XZ: Software, Writing – review & editing.
